# Draft Genome Sequences of Seven *Limosilactobacillus fermentum* Indigenously Isolated Probiotic Strains from the Artisanal Fermented Milk Product Dahi

**DOI:** 10.1128/mra.00742-22

**Published:** 2022-10-10

**Authors:** Muhammad Nadeem Khan, Muhammad Imran

**Affiliations:** a Department of Microbiology, Quaid-i-Azam University, Islamabad, Pakistan; University of Maryland School of Medicine

## Abstract

Here, we report the draft genome sequences of seven strains of potentially probiotic Limosilactobacillus fermentum isolated from the traditional fermented milk product dahi. The estimated average genome size was 1,955,815 bp, with a median GC content of 52%. Genome annotation predicted an average of 1,871 protein-coding genes and 47 RNAs.

## ANNOUNCEMENT

Lactic acid bacteria (LAB) are known for their ability to impart a multitude of health benefits. Recently, Lactobacillus fermentum, a member of LAB, was reclassified as Limosilactobacillus fermentum for its metabolic and ecological properties (1). L. fermentum is a Gram-positive, non-spore-forming, nonmotile, rod-shaped bacterium that can grow singly, in pairs, or in short chains (1). *L. fermentum* is usually found in habitats that are nutrient rich and are associated with humans, plants, animals, and food (2). *L. fermentum* is considered a promising potential probiotic candidate due to its lack of antibiotic resistance genes, along with its antioxidative, antimicrobial, and cholesterol reduction properties (3–5). The genome of *L. fermentum* possesses specific genes that help it to tolerate the immune system of the host, interact with the other microbes of the gut, and colonize the host epithelium (6–8).

We report the genome sequences of seven strains of *L. fermentum* (QAULFN56, QAULFN64, QAULFN21, QAULFN53, QAULFN54, QAULFN55, and QAULFN62) that were isolated from the traditional fermented milk product dahi. Samples were collected from dairy corner shops of Rawalpindi, Islamabad, Pakistan. For isolation, 100 μl of dahi sample was plated on de Man Rogosa Sharpe (MRS) agar (pH 6.8 ± 2) and incubated at 37°C for 24 h in a GasPak anaerobic system (Sigma-Aldrich). Single colonies from MRS plates were subcultured, and purified cultures were obtained. The cultures were identified as strains of *L. fermentum* with a microbial identification system explained previously (9), followed by analysis using the ON-rep-seq gene sequencing tool (10).

The pure colonies from agar plates were grown overnight in MRS broth followed by DNA extraction using InstaGene matrix (Bio-Rad Laboratories, California, USA). A Qubit 2.0 fluorometer (Invitrogen, Carlsbad, CA, USA) was used to quantify the DNA. DNA libraries for high-throughput sequencing were obtained with the commercially available Vazyme TruePrep DNA library prep kit V2 (Vazyme Biotech, Nanjing, China). Sequencing of these libraries was performed on an Illumina HiSeq-2000 sequencing platform (BGI-Shenzhen, Shenzhen, China) with an average read length of 101 bp. FASTQ files were generated, and average total raw reads were 10,294,243. Default parameters were used for all software unless otherwise specified. The quality trimming of the sequencing reads was performed using Trimmomatic version 0.38 (11). The number of filtered average reads was 9,340,781, and these were aligned with the reference genome of *L. fermentum* IFO 3956 (NCBI RefSeq accession number NC_010610). The assembly of the high-quality reads was performed using the Velvet (v1.2.10) assembler (12). The *de novo* assembly results were evaluated using Quast software (13). The assembled genomes were annotated through PGAP (14). The subsystem identification in the genomes was determined through RAST (15), while evaluation of orthologous genes was performed via clusters of orthologous genes (COGs) analysis (16) and eggNOG (17). The results are presented in Table 1. The genomes were mined for the presence of bacteriocin, antibiotic resistance, and CRISPR/CRISPR-Cas genes through the online tools BAGEL4, CARD, and CRISPR Finder, respectively (18–22). Except for strains QAULFN54 and QAULFN21, the rest of the five strains had bacteriocin genes. CRISPR sequences were identified in all the strains except QAULFN54. The presence of CRISPRs suggested immunity to foreign attacking agents, like phages, jumping genes, or transfer elements (23).

**TABLE 1:** Genome statistics for *L. fermentum* isolates obtained from the fermented milk product dahi

Feature	QAULFN56	QAULFN64	QAULFN21	QAULFN53	QAULFN54	QAULFN55	QAULFN62
GenBank accession no.	JAJAOT000000000	JAJAOV000000000	JAJAOP000000000	JAJAOQ000000000	JAJAOR000000000	JAJAOS000000000	JAJAOU000000000
SRA accession no.	SRR19959948	SRR19959946	SRR19959952	SRR19959951	SRR19959950	SRR19959949	SRR19959947
Raw reads	10,296,951	10,294,952	10,293,176	10,292,199	10,297,350	10,292,921	10,292,151
Genome coverage	300×	300×	300×	300×	300×	300×	300×
Size (bp)	1,900,053	1,900,053	1,867,005	2,077,539	1,966,551	2,011,311	1,968,193
% GC	52.4	52.4	52.8	51.7	52	51.8	52
Contigs	102	102	74	170	129	137	80
Total genes	1,919	1,919	1,848	2,148	2,059	2,041	2,001
CDSs with proteins	1,803	1,803	1,724	2,002	1,965	1,935	1,869
RNAs	52	52	63	53	20	29	60
Pseudogenes	64	64	61	80	74	70	72
Subsystem	207	207	212	220	215	220	213
COGs	776	778	795	845	826	840	822
eggNOG features	1,653	1,643	1,595	1,800	1,715	1,749	1,684
Bacteriocin gene	Enterolysin_A	Enterolysin_A	Enterolysin_A	Enterolysin_A	Enterolysin_A	Enterolysin_A	Enterolysin_A
CRISPR^*a*^	C	C	C	P	Nil	C	C

a P, possible; C, confirmed. No resistance gene was found in any of the strains.

### Data availability.

The genomic data reported here have been deposited in GenBank under the accession numbers given in Table 1. The SRA accession numbers are also provided in Table 1. The BioProject number is PRJNA744373, and the relevant BioSample numbers are SAMN20114169 to SAMN20114175.
